# Modeling Skewness in Human Transcriptomes

**DOI:** 10.1371/journal.pone.0038919

**Published:** 2012-06-11

**Authors:** Joaquim Casellas, Luis Varona

**Affiliations:** 1 Departament de Ciència Animal i dels Aliments, Universitat Autònoma de Barcelona, Bellaterra, Spain; 2 Departamento de Anatomía, Embriología y Genética Animal, Universidad de Zaragoza, Zaragoza, Spain; King Abdullah University of Science and Technology, Saudi Arabia

## Abstract

Gene expression data are influenced by multiple biological and technological factors leading to a wide range of dispersion scenarios, although skewed patterns are not commonly addressed in microarray analyses. In this study, the distribution pattern of several human transcriptomes has been studied on free-access microarray gene expression data. Our results showed that, even in previously normalized gene expression data, probe and differential expression within probe effects suffer from substantial departures from the commonly assumed symmetric Gaussian distribution. We developed a flexible mixed model for non-competitive microarray data analysis that accounted for asymmetric and heavy-tailed (Student’s *t* distribution) dispersion processes. Random effects for gene expression data were modeled under asymmetric Student’s *t* distributions where the asymmetry parameter (λ) took values from perfect symmetry (λ = 0) to right- (λ>0) or left-side (λ>0) over-expression patterns. This approach was applied to four free-access human data sets and revealed clearly better model performance when comparing with standard approaches accounting for traditional symmetric Gaussian distribution patterns. Our analyses on human gene expression data revealed a substantial degree of right-hand asymmetry for probe effects, whereas differential gene expression addressed both symmetric and left-hand asymmetric patterns. Although these results cannot be extrapolated to all microarray experiments, they highlighted the incidence of skew dispersion patterns in human transcriptome; moreover, we provided a new analytical approach to appropriately address this biological phenomenon. The source code of the program accommodating these analytical developments and additional information about practical aspects on running the program are freely available by request to the corresponding author of this article.

## Introduction

Mixed models have been advocated in gene expression analyses due to their superiority in partitioning sources of variation and their flexibility for accommodating various experimental designs [1]; furthermore, they can be used for joint analysis of all loci [2], appropriately accounting for variability both across and within microarray probes [3]. Microarray data sets are characterized by high dimensionality in the sense of a small number of replicates (i.e. microarray slides) and a large number of probes per replicate. Mixed models account for these peculiarities of the microarray gene expression data, the sources of variation being preferentially treated as random effects [4] to appropriately address large numbers of levels with scarce amounts of information per level. A typical assumption for the distribution of random effects in mixed model analyses is the Gaussian density function [4], which is systematically applied in standard gene expression analyses [3], [5]. Although this parametric assumption could be viewed as a reasonable compromise between mathematical convenience and biological plausibility, its suitability in gene-expression analyses has been questioned in recent studies [6]–[12].

Taking the probe-specific differential expression effect between two treatments, the Gaussian distribution forces a symmetrical pattern between the two treatments, whereas a wide range of skewed distributions and treatment-related over-expressions may seem more reasonable. Moreover, the Gaussian assumption suffers substantial misadjusts in the presence of outliers [13], which are common in microarray data [14]. Given the inconsistencies of the Gaussian distribution for random effects in the gene expression data, recent researches have proposed parametric alternatives for modeling gene expression data, assuming heavy-tailed processes like Cauchy [8] and Student’s *t* distributions [7] or asymmetric distributions like Pareto [15], Gamma [6] and skew Laplace [16], [17]. Although these studies have reported substantial improvements in terms of model fit to experimental data, none of them allowed joint, flexible modeling of gene expression data under variable incidence of outliers or asymmetry, or the incidence of both positive (right-hand tail over-expressed) and negative (left-hand tail over-expressed) asymmetric patterns.

The Student’s *t* distribution has been proposed as a useful assumption for attenuating the impact of outliers in mixed models [7] and, furthermore, asymmetry can be easily accommodated in the Student’s *t* density [18]. Within this context, the aim of this research was to check for asymmetric and heavy-tailed patterns in random effects of gene expression data, developing a new analytical approach to appropriately accommodate both sources of departure from the standard symmetric Gaussian assumption.

## Results

### Model Comparison

Four independent microarray gene expression data sets from human tissues (Table 1) were analyzed under three different hierarchical mixed linear models. These analyses accounted for the systematic effect of each microarray slide and two random sources of variation, probe and treatment within probe (with two levels in each data set). Models differed in the a priori distributions of these random effects, they being multivariate normal densities (Model SG) [5], symmetric Student’s *t* densities (Model ST), or asymmetric Student’s *t* densities (Model AS) following Sahu et al. [18]. The deviance information criterion (DIC) [19] assessed model performance under these three different prior distributions for random effects, revealing a huge penalization for model SG in all cases (Table 2). Note that models with a smaller DIC were favored as this indicated a better fit and lower degree of model complexity [19]. In all four comparisons, DIC sequentially and drastically reduced with models ST and AT (Table 2), where Sahu et al. [18] asymmetric Student’s *t* priors for probe and differential expression within probe effects were clearly preferred (Table 2). Note that differences larger that 3 to 5 DIC units are assumed as statistically relevant [19] and Model AT showed the lowest DIC with 4,678 (dataset 1) to 60,335 (dataset 3) less DIC units than Model ST. These large DIC departures ruled out any possible controversy concerning the most preferable model. It is important to note that the number of differentially expressed genes reduced with Model AT, also suggesting a more conservative behavior for this parameterization (Table 2).

**Table 1:** Summary of the free-access data sets analyzed.

	Platform(a)	Tissue	Groups of comparison (number ofsamples per group)	Reference	GEO(b)
Dataset 1	Affymetrix GeneChip Human FullLength Array HuGeneFL	Mononuclear cell layer	Non-pulmonary arterial hypertension (6)*vs*. pulmonary arterial hypertension (14)	Bull et al. [26]	GSE703
Dataset 2	Affymetrix GeneChip Human FullLength Array HuGeneFL	Bronchoalveolar lavage cells	Non-smoker (5) *vs*. smoker (5)	Heguy et al. [27]	GSE3212
Dataset 3	Affymetrix GeneChip Human GenomeU133 Plus 2.0 Array	Spermatozoa	Normal (12) *vs.* teratozoospermicindividuals (8)	Platts et al. [28]	GSE6969
Dataset 4	Illumina humanRef-8 v2.0 expressionbeadchip	Carotid endarterectomy samples	Carotid artery stenosis treated withmycophenolate (9) *vs*. placebo (11)	Unpublished	GSE13922

(a) The approximate number of interrogated transcripts were 5,000, 47,000 and 16,000 for Affymetrix GeneChip Human Full Length Array HuGeneFL (Affymetrix, Inc., Santa Clara, CA), Affymetrix GeneChip Human Genome U133 Plus 2.0 Array (Affymetrix, Inc., Santa Clara, CA) and Illumina human Ref-8 v2.0 expression beadchip (Illumina, Inc., San Diego, CA), respectively.

(b) Gene Expression Omnibus accession number (http://www.ncbi.nlm.nih.gov/geo/).

**Table 2:** Model comparison and characterization of the dispersion patter of probe and differential expression within probe under Model AT.

	Dataset 1	Dataset 2	Dataset 3	Dataset 4
DIC(a) (and number of probes with significant differential expression(b))				
Model SG^(c)^	284,161 (189)	231,581 (5)	3,692,344 (702)	1,756,122 (12)
Model ST^(d)^	247,509 (31)	224,741 (1)	3,614,823 (692)	1,734,053 (4)
Model AT^(e)^	242,831 (2)	188,835 (0)	3,554,488 (639)	1,724,667 (2)
Parameters(f) under Model AT. Mode (and highest posterior density region at 95%)				
*v_p_*	8.95 (4.21 to 26.61)	5.62 (4.16 to 11.05)	6.77 (4.15 to 16.0)	8.87 (4.40 to 30.09)
λ*_p_*	0.38 (0.04 to 0.66)	0.13 (0.01 to 0.32)	1.84 (1.61 to 1.93)	2.03 (1.98 to 2.09)
*v_d_*	7.36 (4.18 to 18.05)	6.90 (4.15 to 19.76)	5.99 (4.38 to 11.51)	8.48 (4.66 to 23.90)
λ*_d_*	0.01 (−0.04 to 0.06)	−0.00 (−0.04 to 0.04)	−1.88 (−1.96 to −1.81)	−0.00 (−0.01 to 0.01)

(a) Deviance information criterion.

(b) Differentially expressed genes after Bonferroni [29]-like correction (α = 0.05). The adjusted significance threshold for posterior probabilities was calculated as α/π, were π was the number of probes included in each analysis.

Random effects **g** and **d**(**g**) were assumed as symmetric Gaussian^(c)^, symmetric Student’s *t* ^(d)^ or asymmetric Student’s *t* ^(e)^ distributed following Sahu et al. [18].

(f) Degrees of freedom (*v*) and asymmetry parameter (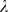) for probe (*p*) and differential expression within probe (*d*) effects.

### Estimates for Asymmetry and non-Gaussian Patterns

**Figure 1:**
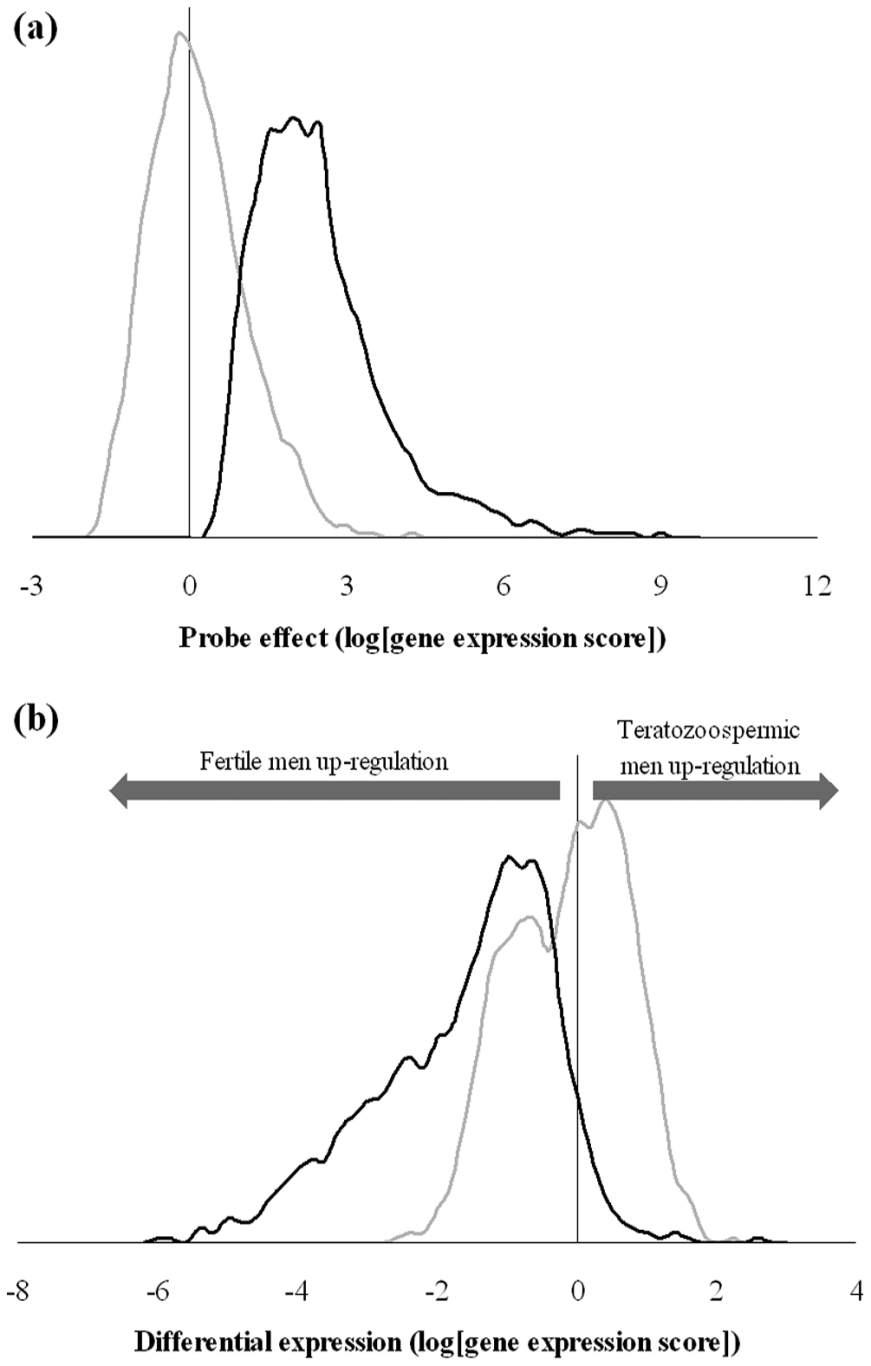
Distribution of mean estimates for probe (**a**) and differential expression within-probe (**b**) effects under Model SG (grey line) and Model AT (black line) for data set 3.

Under Model AT, the non-Gaussian distributions of probe and differential expression within probe effects were characterized in terms of heavy tails (Student’s *t*) and asymmetric dispersion patterns by means of *v* (degrees of freedom of the Student’s *t* distribution) and 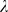 (asymmetry parameter). Probe effects revealed both heavy tails and positive asymmetry with a substantial over-expression of the right tail of the distribution. The modal estimates of the degrees of freedom fluctuated between 5.62 (data set 2) and 8.95 (data set 1), with the highest posterior density region at 95% roughly ranged between 4 and 30. The right-hand asymmetry was clearly demonstrated in all datasets with positive modal estimates of 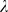, their HPD95 excluding the null or negative values (Figure 1a). The differential expression within probe effect showed a similar pattern with small *v*, although significant asymmetry was only revealed in data set 3 (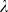 = −1.88; HPD95: −1.96 to −1.81; Figure 1b).

## Discussion

The asymmetric and non-Gaussian distribution of the human transcriptome has been revealed in four independent human data sets from different microarray platforms and technologies. Although our results cannot be completely extrapolated to all microarray data, they show that deviations from the standard Gaussian prior for random effects should be accurately considered in current gene expression studies. Normalization of gene expression data has been a topic of main interest during the last decade [20], [21], but our results suggested that non-Gaussian patterns must be considered as an inherent property of gene expression data, and this phenomenon should be appropriately accounted for in analytical models in order to avoid biases on final estimates (Table 2). Note that the Student’s *t* density converges to a Gaussian density when *v* tends to infinity, although both densities are assumed roughly similar for *v* values larger than 30 [13]. In our case, small modal estimates (<10) were obtained for the degrees of freedom of the Student’s *t* distribution, suggesting a relevant departure from the standard Gaussian distribution as corroborated by the DIC statistic. Our small values for *v* reported a substantial incidence of outlier gene expressions as was previously sugested by Gottardo et al. [7] and Khondoker et al. [8] in alternative microarray data sets. Moreover, Model AT was preferred, highlighting the usefulness of the hierarchical mixed model with asymmetric Student’s *t* prior distributions for random sources of variation.

All data sets agreed with right-hand over-distributed probe effects, whereas left-hand over-expression was revealed for differential expression within probe estimates in data set 3 (Figure 1b). This right-hand asymmetry in human transcriptome must be linked to the fact that lowly expressed probes are roughly grouped in the left tail of the scanning spectrum due to technological limitations of the microarray technique, whereas a substantial incidence of high or extremely-high gene expression intensities can be anticipated [22]. Note that this phenomenon is not commonly accounted for in gene expression analyses worldwide, whereas the mixed model parameterization developed in this manuscript provides a highly flexible statistical tool accounting for the non-Gaussian properties of human (and even non-human) transcriptome. As shown in Figure 1a, departures from the standard Model SG do not reduce to the symmetry pattern only, but also rely on the average mathematical expectation for probe effects. Sahu’s et al. [18] method can accommodate distributions with non-zero modal estimates (Figure 1a). Focusing on data set 3, the modal estimate for differential expression effects was placed around 2 (Model AT) and linked to larger estimates for the array effect when comparing with Model SG. It implied a moderate relocation of systematic and random sources of variation for gene expression data in the output of the mixed model.

Results from differential expression within probe effects highlighted the remarkable flexibility of Sahu’s et al. [18] method to accommodate any kind of asymmetry pattern. This peculiarity is of special relevance for differential gene expression given that a wide range of asymmetric patterns could be find in gene expression studies. Although the standard symmetric Gaussian distributions may be valid sometimes, a wide range of left- and right-tail over-expressions could be addressed with Model AT. Indeed, data set 3 (all datasets) showed that asymmetric (heavy-tailed) patterns are not unusual and they must be considered in gene expression analyses. The relevance of a proper modeling of random effects is clearly highlighted in Figure 1b where the symmetric Gaussian prior distribution for the differential expression produces a bimodal artifact in the posterior distribution of the estimates, clearly differing from the expected drawn under the a priori assumption.

Note that all model comparisons were made on the basis of the DIC statistic [19], a widely used statistical criterion to assess model complexity and fit. Indeed, DIC measures posterior predictive error by penalizing the fit of the model (i.e., deviance) by its complexity, determined by the effective number of parameters as defined by Spiegelhalter et al. [19]. Within this context, model AT must be clearly viewed as the most parsimonious and reliable parameterization, at least among the alternatives we are considering in this study. DIC evidenced that the incidence of asymmetry and heavy-tailed patterns in human gene expression data must be out of any doubt and, as consequence, model AT characterized a quasi-optimum approach to analyze this kind of microarray data. Nevertheless, DIC does not provide specific information about testing properties of current models when evaluating differentially expressed genes, although better model fit must be linked to better testing properties. Model AT reported the smallest number of differentially expressed probes in all data sets (Table 2) and all those probes were previously identified as differentially expressed by models SG and ST. The same patterns were obtained in preliminary analyses of simulated gene expression data (results not shown) and suggested that the better fit and more restrictive testing behavior of model AT could be linked to false positives under models SG and ST.

In conclusion, the incidence of asymmetric random effects has been highlighted in non-competitive gene expression data from human tissues; the new model proposed below provides a better adjustment of gene expression data and even a more conservative testing pattern has been suggested. Although this manuscript has focused on non-competitive hybridization microarrays, models can be easily adapted to two channel microarrays following Purdom and Holmes [16].

## Materials and Methods

### Mixed Model for Non-competitive Microarray Data

We assume as a starting point non-competitive hybridization microarray data from *n* unrelated individuals appropriately grouped in two different treatments (e.g. normal versus tumor cells) and *m* probes. These data (**y**) can be analyzed by the mixed model:
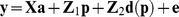
where **X**, **Z**_1_, and **Z**_2_ are incidence matrices for array (**a**), probe (**p**) and differential expression (between treatments) within probe (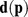) effects, and **e** is the vector of residuals. Following a standard Bayesian development, the joint posterior distribution of all parameters in the model conditional to the data is proportional to the Bayesian likelihood,



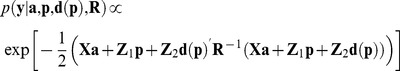
multiplied by the *a priori* distribution of each parameter in the model. Note that this equation describes a heteroskedastic normal density [5] with gene-specific residual variances and with null residual covariance between genes (**R**). *A priori* distributions for **p** and 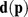 could be described as



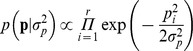
and



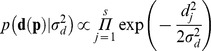
they being independent Gaussian densities with a mean of zero and variances equal to 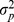 and 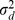, respectively (Model SG). Note that *i* was the number of elements in **p** and *j* was the number of elements in **d**(**p**). Nevertheless, robustness must be gained under a skew-Student’s *t* prior. This prior can be parameterized as a skewed-normal density,



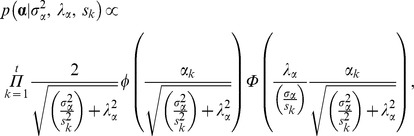
multiplied by the conditional distribution of the mixing parameter (*s_k_*), this being a Gamma prior,



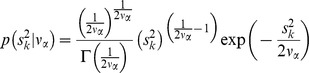
Note that 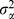 was the scale parameter, 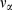 were the degrees of freedom, and 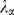 was the asymmetry parameter modelled following Sahu et al. [18] (Model AT). Moreover, φ and 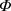 denoted the density function and cumulative distribution function of a standard normal distribution with kernel as defined between parentheses, respectively, and 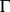 was the standard gamma function with argument as defined within parentheses. Note that 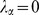 describes perfect symmetry, whereas right (or left) tail proportionally increases for positive (or negative) values of 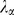. An uniform prior distribution was defined for 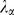, as previously suggested by Varona et al. [23]. Symmetric Student’s *t* priors (Model ST) can be easily defined if 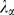 is appropriately fixed to 0. *A priori* distributions for degrees of freedom were defined as exponential [24] and flat priors were assumed for the remaining parameters. Note that the Student’s *t* density converges to the Gaussian one when degrees of freedom tend to infinity, whereas few degrees of freedom account for heavy-tailed densities [13]. All the unknown factors in the model can be easily sampled from their joint posterior distribution by Markov chain Monte Carlo methods [25].

### Example with Free-access Human Gene Expression Data

To illustrate the asymmetric pattern of the human transcriptome, we applied the models to four free-access human microarray datasets (http://www.ncbi.nlm.nih.gov/geo/; accession numbers GSE703, GSE3212, GSE6969 and GSE 13922). Note that all data sets are MIAME compliant and they were previously deposited in the Gene Expression Omnibus database (http://www.ncbi.nlm.nih.gov/geo/). These datasets were representative of two different trademarks and hybridization technologies, evaluated in diverse human tissues (see Table 1). All of them focused on the comparison between two groups, non-pulmonary arterial hypertension *versus* pulmonary arterial hypertension (Dataset 1; [26]), non-smoker *versus* smoker (Dataset 2; [27]), normal *versus* teratozoospermic individuals (Dataset 3; [28]) and carotid artery stenosis treated with mycophenolate *versus* placebo (Dataset 4; unpublished). A base 2 logarithm was applied to normalize gene-expression scores.

Note that the four human data sets were selected at random to evaluate the three mixed model parameterizations on different human tissues and microarray platforms. Of course, both tissue and data quality could have some impact on the distribution pattern, although this escaped from the objectives of this research. Different preprocessing approaches would have different impacts on further analyses of gene expression data and even skewed or heavy-tailed patterns could be partially addressed by preliminary data editing methodologies such as normalization of background correction. Nevertheless, we focused on the development, implementation and evaluation of a reliable parameterization to account for non-Gaussian patterns in gene expression data, assuming that all preliminary data editing processes where properly satisfied.

For each dataset, the three different models were analyzed (models SG, ST and AT). Each model was solved through Bayesian inference with a single Monte Carlo Markov chain of 500,000 elements after discarding the first 50,000 as burn-in. Models were compared with the DIC [19].
